# Ruxolitinib synergistically enhances the anti-tumor activity of paclitaxel in human ovarian cancer

**DOI:** 10.18632/oncotarget.24368

**Published:** 2018-01-31

**Authors:** Ernest S. Han, Wei Wen, Thanh H. Dellinger, Jun Wu, Selena A. Lu, Richard Jove, John H. Yim

**Affiliations:** ^1^ Department of Surgery, Beckman Research Institute, City of Hope Comprehensive Cancer Center, Duarte, CA 91010, USA; ^2^ Department of Molecular Medicine, Beckman Research Institute, City of Hope Comprehensive Cancer Center, Duarte, CA 91010, USA; ^3^ Department of Comparative Medicine, Beckman Research Institute, City of Hope Comprehensive Cancer Center, Duarte, CA 91010, USA; ^4^ Current/Present address: Cell Therapy Institute, Nova Southeastern University, Fort Lauderdale, FL 33314, USA

**Keywords:** ruxolitinib, ovarian, paclitaxel, combination, synergy

## Abstract

Treatment for ovarian cancer remains challenging despite a high initial response rate to first line platinum-taxane treatment. Most patients eventually experience recurrence and require further treatment. Persistent activation of STAT3 is associated with cancer growth and progression and is also involved in cell resistance to platinum and taxane treatment. Targeting JAK/STAT3, therefore, could be a potential novel therapeutic approach for treating advanced and chemoresistant ovarian cancer. We investigated the therapeutic potential of ruxolitinib, a JAK1/JAK2 inhibitor that has been FDA-approved for the treatment of myelofibrosis, to treat ovarian cancer either alone or in combination with conventional chemotherapy agents. We show that ruxolitinib inhibits STAT3 activation and ovarian tumor growth both in ovarian cancer cells and in an ovarian cancer mouse model. In addition, ruxolitinib significantly increases the anti-tumor activity of chemotherapy agents, including paclitaxel, cisplatin, carboplatin, doxorubicin and topotecan in ovarian cancer cells. Evaluation of the combination index (CI) shows that ruxolitinib synergistically interacts with paclitaxel in all three human ovarian cancer cells. Finally, our results demonstrate that combination of ruxolitinib and paclitaxel leads to a greater reduction of tumor growth compared to single treatment of either agent in a tumor mouse model that represents late stage ovarian cancer with peritoneal metastasis and ascites formation. Taken together, our findings provide a foundation for clinical trials with ruxolitinib, either as a single agent or in combination with paclitaxel, for the treatment of recurrent and advanced ovarian cancer.

## INTRODUCTION

Treatment for ovarian cancer remains challenging despite a high initial response rate to first line platinum-taxane treatment. Most patients eventually experience recurrence and require further treatment. However, there is no effective treatment for recurrent drug-resistant ovarian cancer. Despite efforts to overcome resistance using alternate chemotherapy agents, mortality remains high in platinum-resistant patients [1–7]. Therefore, there is a critical need to develop novel strategies to treat advanced and drug resistant ovarian cancer.

STAT3, a promising molecular target for cancer therapies, is a member of the STAT family of transcription factors that mediate cellular responses to cytokines and growth factors. In healthy tissue, STAT3 is predominantly located in the cytoplasm in an inactive form. In response to cytokine stimulation, STAT3 is phosphorylated at Tyr705 by Janus family kinases (JAK) [8, 9]. Phosphorylated STAT3 protein can translocate into the nucleus, bind to DNA, and activate the transcription of various genes that regulate vital cellular functions, including cell survival, proliferation, angiogenesis, and tumor evasion [10]. Normally, the activation of STAT3 by JAK occurs transiently and is tightly regulated. However, in cancer cells STAT3 is constitutively activated [10–14], and its persistent activation is associated with a poor prognosis in cancer patients, including ovarian cancer patients [15, 16].

Several recent studies have demonstrated a critical role of STAT3 in ovarian cancer growth and progression. Inhibition of STAT3 activation has led to reduced tumor growth, decreased peritoneal dissemination, and diminished ascites production in a peritoneal ovarian tumor model [17–19]. In addition, emerging evidence suggests that activation of STAT3 is involved in resistance to both receptor tyrosine kinase -target therapy and conventional chemotherapy [20–26]. In addition, increased STAT3 activation occurs in paclitaxel-resistant ovarian cancer cells, and STAT3 inhibition potently increases anti-tumor activity of paclitaxel [27–29]. Targeting JAK1/STAT3, therefore, could be a potential novel therapeutic approach for treating advanced and chemoresistant ovarian cancer.

Ruxolitinib is a potent and selective oral JAK1 and JAK2 inhibitor that was FDA-approved in 2011 for the treatment of myelofibrosis (MF), post-polycythemia vera myelofibrosis (PPV-MF), and post-essential thrombocythemia myelofibrosis (PET-MF) [30–32]. The therapeutic potential of ruxolitinib in solid tumors is currently undergoing clinical evaluation in ovarian, metastatic breast, and pancreatic cancers [33–35]. However, there is little pre-clinical information available about ruxolitinib in ovarian cancer treatment.

In this study, we investigated the anti-tumor activity of ruxolitinib, either alone or in combination with chemotherapy agents, in human ovarian cancer both *in vitro* and *in vivo*.

## RESULTS

### Effect of ruxolitinib on phosphorylation of STAT3 and cell viability in human ovarian cancer cells

**Figure 1:**
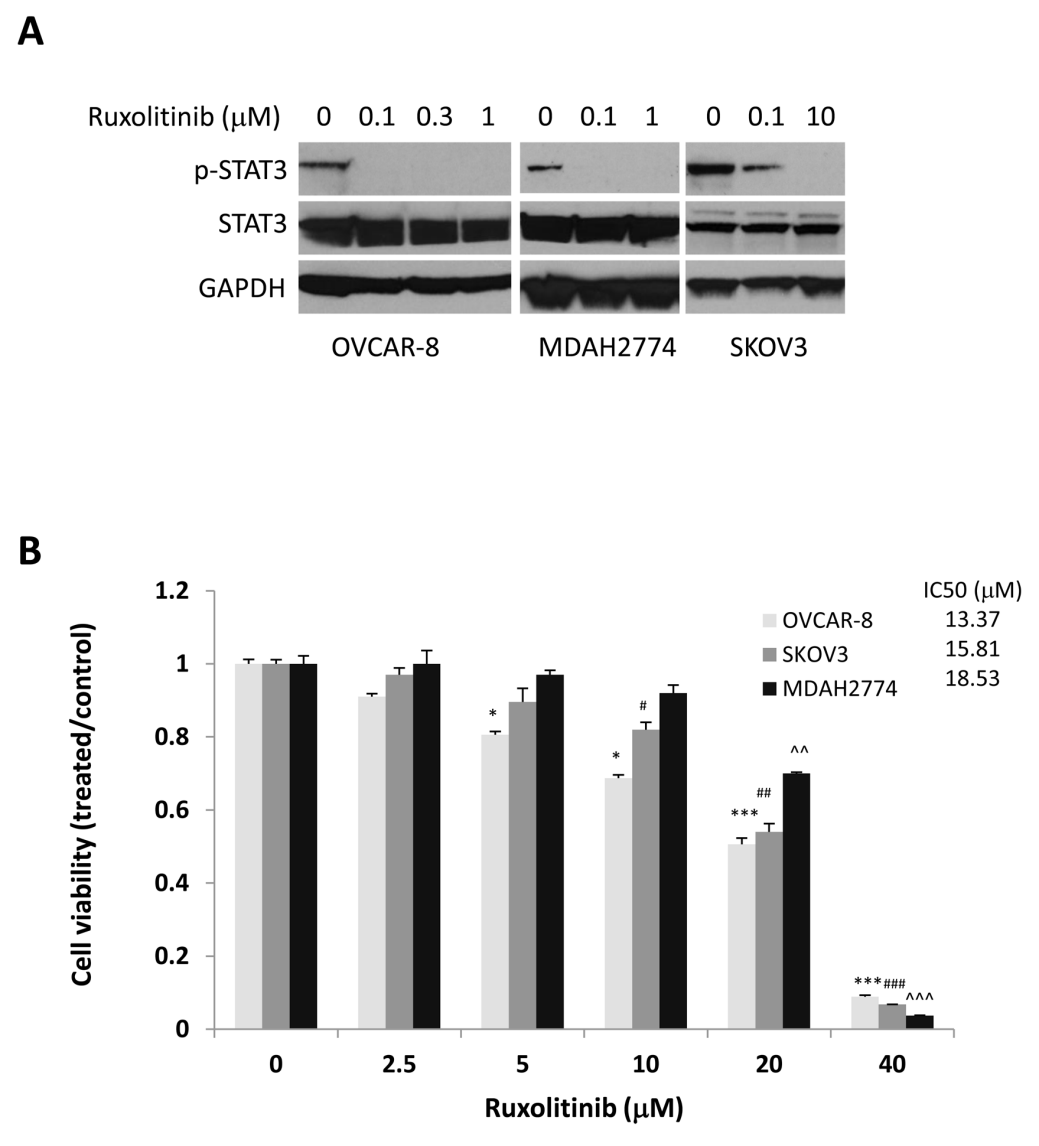
Anti-tumor activity of ruxolitinib in ovarian cancer **(A)** Dose-dependent inhibition of STAT3 phosphorylation. Human ovarian cancer cells, OVCAR-8, MDAH2774, and SKOV3, were treated with the indicated concentrations of ruxolitinib for 24 h. Phosphorylation of STAT3 was analyzed by Western blot. **(B)** Dose dependent inhibition of cell viability. Human ovarian cancer cell lines were treated with the indicated concentrations of ruxolitinib. Cell viability was determined 72 h later. The IC_50_ was determined by the Chou-Talalay method. ^*^P<0.05; ^***^P<0.0005, ruxolitinib *vs* control in OVCAR-8 cells; ^#^*P<*0.05; ^##^P<0.005; ^###^P<0.0005, ruxolitinib *vs* control in SKOV-3 cells; ^^^^P<0.005; ^^^^^P<0.0005, ruxolitinib *vs* control in MDAH2774 cells.

To understand the effect of ruxolitinib on STAT3 phosphorylation, we incubated OVCAR-8, MDAH2774, and SKOV3 human ovarian cancer cells with increasing concentrations of ruxolitinib followed by Western blot analysis. We found that ruxolitinib significantly inhibited phosphorylation of STAT3 in a dose dependent manner in all cells (Figure 1A). To study the anti-tumor activity of ruxolitinib in human ovarian cancer, we first tested the effects of ruxolitinib on the proliferation and viability of OVCAR-8, MDAH2774, and SKOV-3 cells. Cells were incubated with increasing concentrations of ruxolitinib, and cell viability was determined after 72 h. We found that ruxolitinib inhibited cell viability with IC_50_s ranging from 13.37 μM to 18.53 μM (Figure 1B).

**Figure 2:**
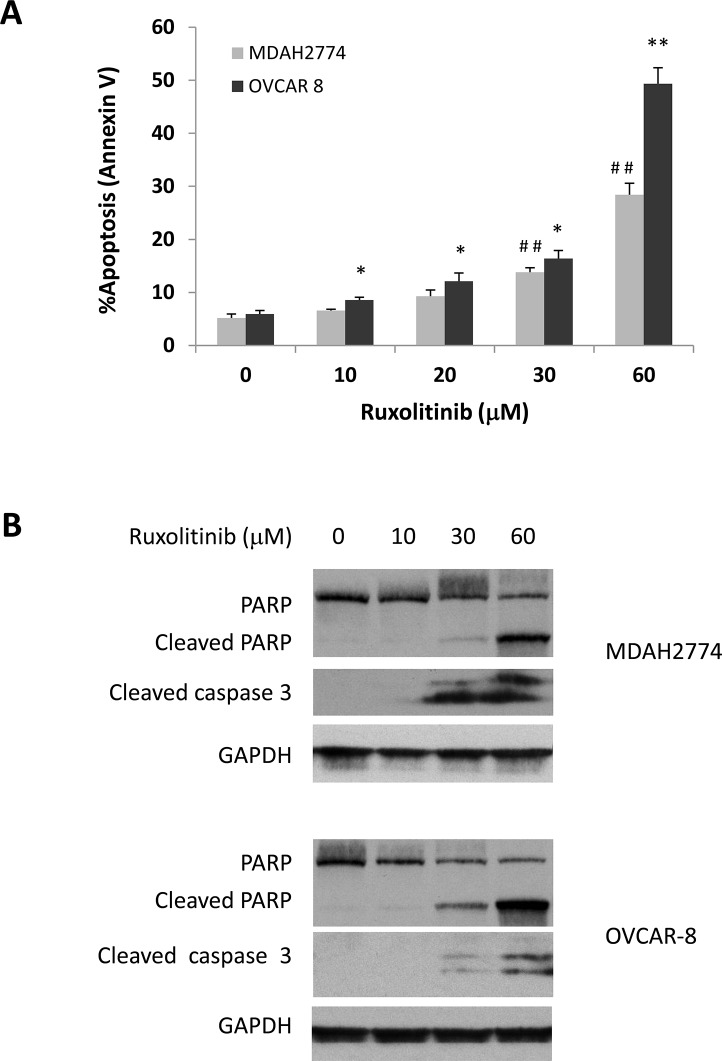
Dose dependent induction of apoptosis **(A)** and **(B)** OVCAR-8 and MDAH 2774 cells were incubated with various concentrations of ruxolitinib for 48 h. Apoptosis was determined by flow cytometry using annexin V and PI staining (A) or using cleaved poly-ADP ribose polymerase (PARP) and cleaved caspase-3 by Western blot (B). ^*^P<0.05; ^**^P<0.005, ruxolitinib *vs* control in MDAH2774 cells; ^##^P<0.005, ruxolitinib *vs* control in OVCAR-8 cells.

Next, to investigate the possibility that reduced cell survival by ruxolitinib could be due to the induction of apoptosis, we treated OVCAR-8 and MDAH2774 cells with various concentrations of ruxolitinib for 48 h. The number of apoptotic cells was then determined by annexin V staining (Figure 2A). We found that ruxolitinib induced cell apoptosis in a dose dependent manner in both OVCAR-8 and MDAH2774 cells. Consistent with the annexin V staining results, generation of cleaved poly-ADP ribose polymerase (PARP), a marker for apoptosis, increased in both OVCAR-8 and MDAH2774 cells treated with ruxolitinib for 48 h (Figure 2B). These results indicate that ruxolitinib could inhibit cell viability of human ovarian cancer cells by promoting apoptosis.

### Effect of ruxolitinib on cell viability induced by chemotherapy agents

**Figure 3:**
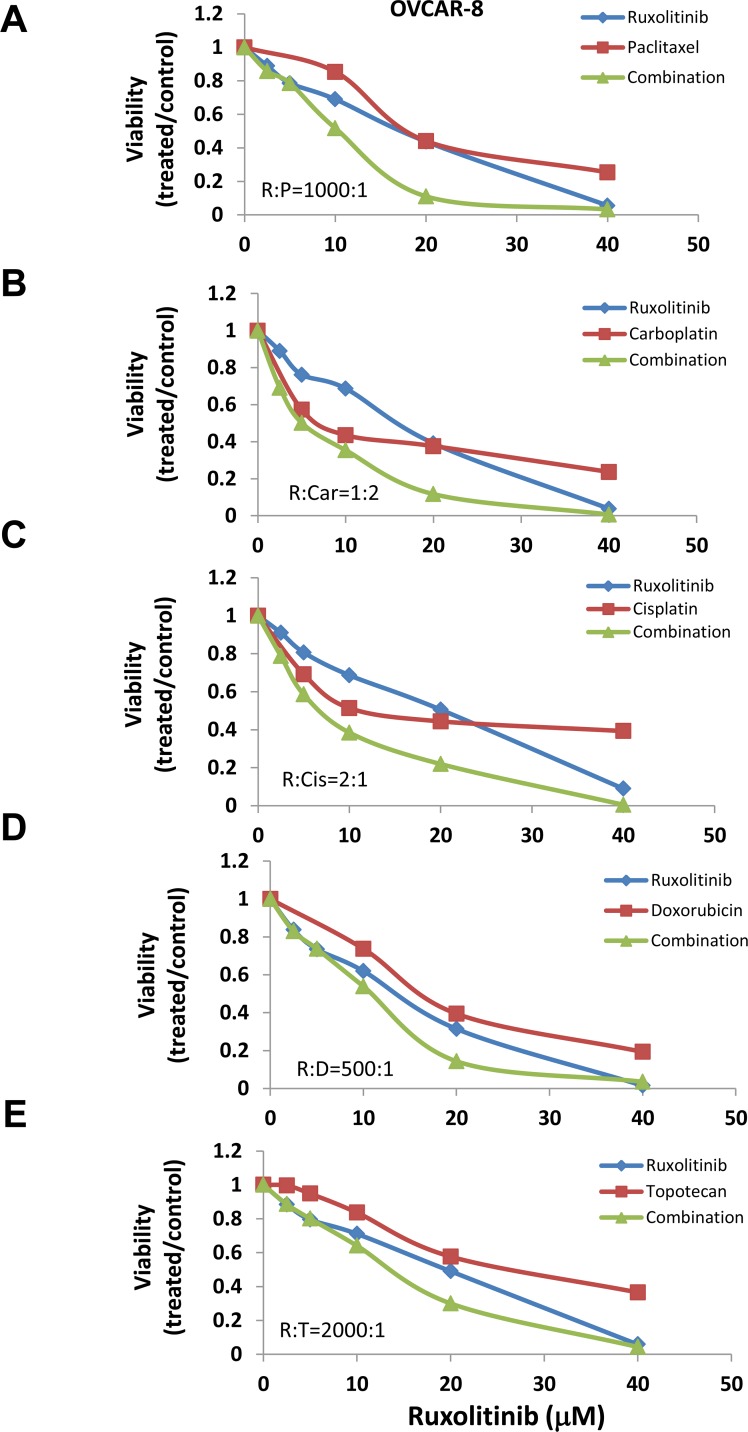
Ruxolitinib enhanced the anti-tumor activity of chemotherapy agents in OVCAR-8 human ovarian cancer cells OVCAR-8 cells were treated with ruxolitinib either alone or together with chemotherapy agents, paclitaxel **(A)**, carboplatin **(B)**, cisplatin **(C)**, doxorubicin **(D)**, and topotecan **(E)**, at various concentrations in a fixed molar ratio. Cell viability was determined 72 h later.

**Figure 4:**
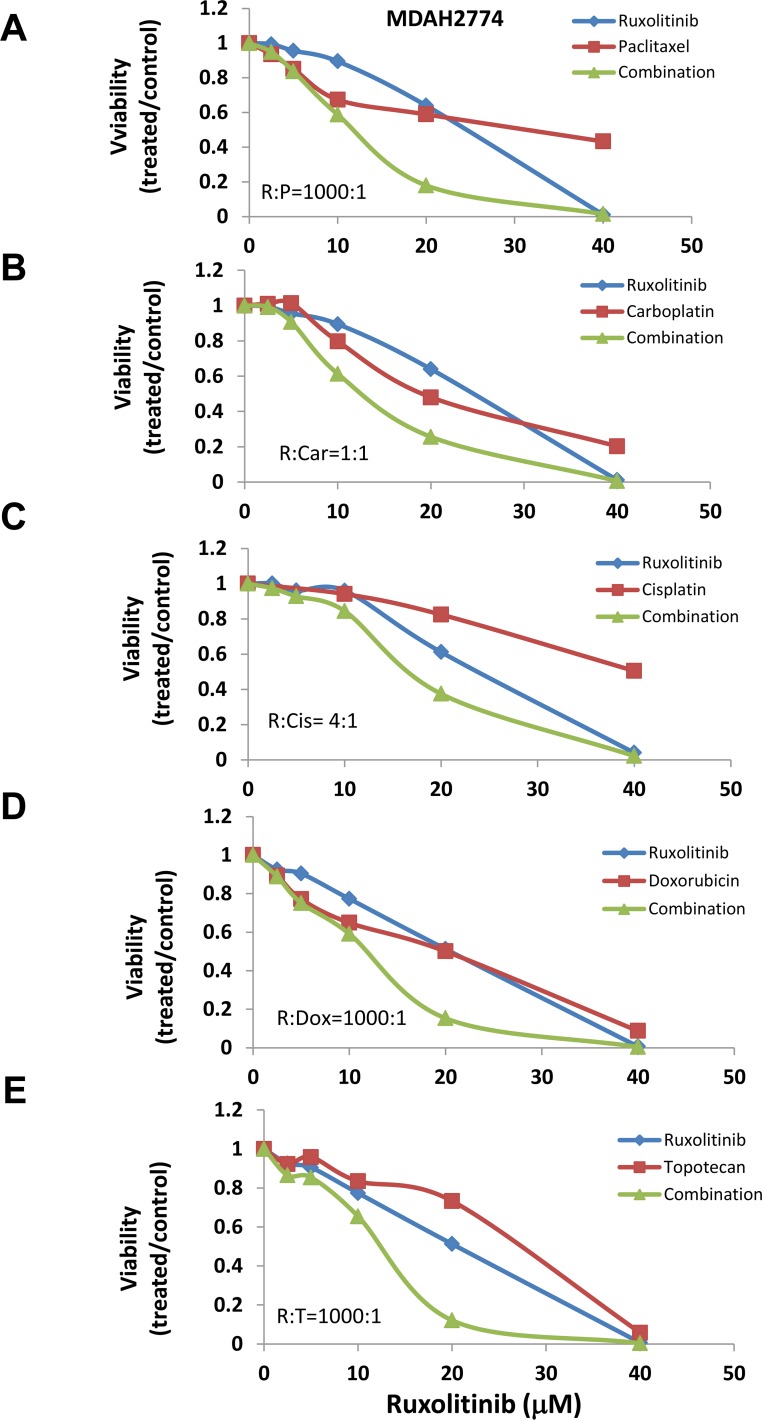
Ruxolitinib enhanced the anti-tumor activity of chemotherapy agents in MDAH2774 human ovarian cancer cells MDAH2774 cells were treated with ruxolitinib either alone or together with chemotherapy agents, paclitaxel **(A)**, carboplatin **(B)**, cisplatin **(C)**, doxorubicin **(D)**, and topotecan **(E)**, at various concentrations in a fixed molar ratio. Cell viability was determined 72 h later.

Previous studies suggest that activation of STAT3 may confer cell resistance to chemotherapy reagents in ovarian cancer cells [20–25]. To understand whether inhibition of the STAT3 pathway could enhance the anti-tumor activity of chemotherapy reagents, we incubated human ovarian cancer cells with several chemotherapy agents, either alone or in combination with ruxolitinib. We found that ruxolitinib significantly increased the anti-tumor activity of paclitaxel, cisplatin, and carboplatin – the first line chemotherapy agents in the treatment of ovarian cancer (Figure 3 and 4). The IC_50_ of paclitaxel was decreased by over two-fold in both OVCAR-8 and MDAH2774 cells (Table 1). Ruxolitinib also increased the anti-tumor activity of doxorubicin and topotecan, commonly used chemotherapy agents for the treatment of relapsed ovarian cancer (Table 1, Figure 3 and 4).

**Table 1:** Ruxolitinib enhanced anti-tumor activity of chemotherapy reagents in human ovarian cancer cells

Cells	Fold reduction														
	Paclitaxel			Carboplatin			Cisplatin			Doxorubicin			Topotecan		
	IC_50_	IC_75_	IC_95_	IC_50_	IC_75_	IC_95_	IC_50_	IC_75_	IC_95_	IC_50_	IC_75_	IC_95_	IC_50_	IC_75_	IC_95_
**OVCAR-8**	2.75	2.90	8.29	1.72	2.51	4.71	2.52	9.49	88.06	2.20	4.13	11.97	1.54	1.63	1.79
**MDAH2774**	2.89	5.36	14.97	1.94	2.23	2.81	2.32	3.3	5.93	1.59	2.26	3.96	2.31	2.83	3.95

**Figure 5:**
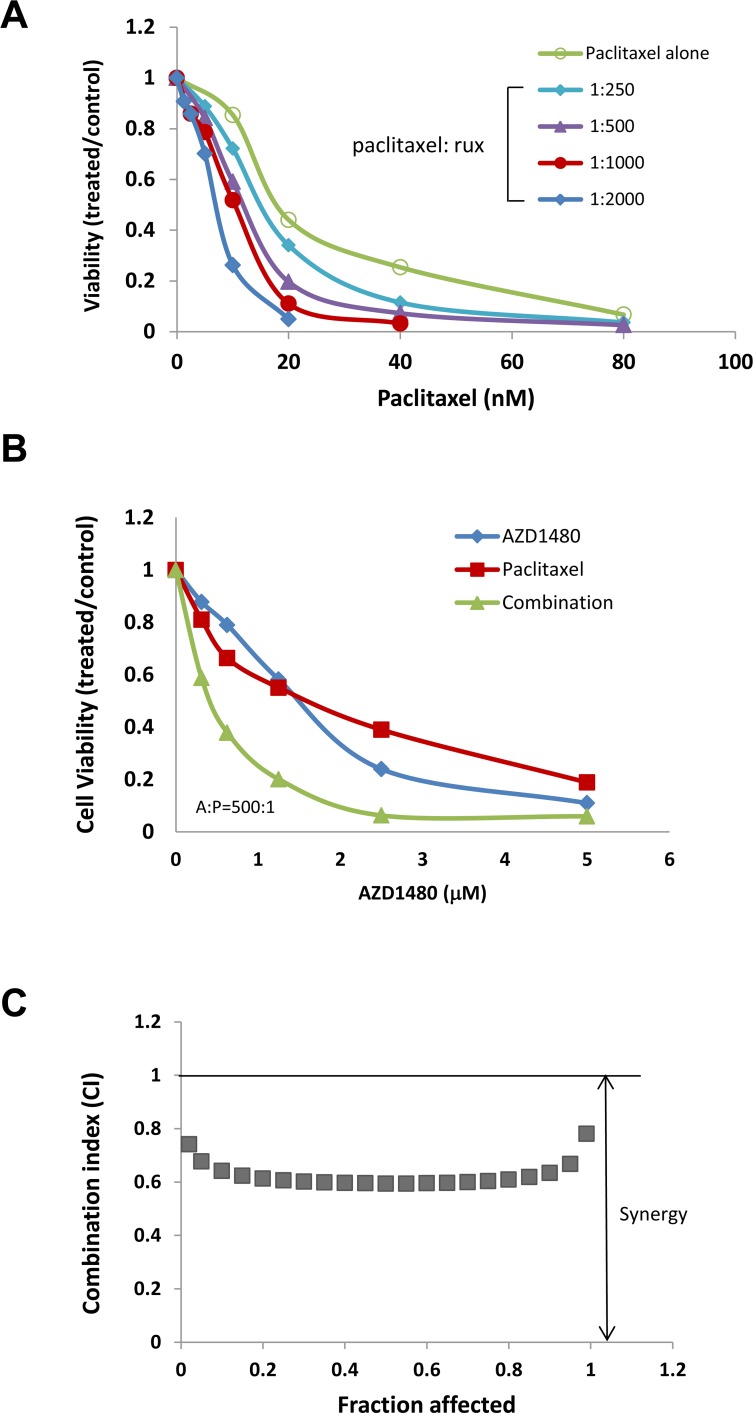
JAK/STAT3 inhibitors enhanced the anti-tumor activity of paclitaxel in human ovarian cancer cells **(A)** OVCAR-8 cells were treated with ruxolitinib either alone or together with paclitaxel in variety of molar ratios. Cell viability was determined 72 h later. **(B)** and **(C)** MDAH2774 cells were treated with AZD1480 (another JAK/STAT3 inhibitor) and paclitaxel, either alone or together. Cell viability was determined 72 h later. CIs (combination index) were determined by the Chou-Talalay method.

To understand whether the increased activity was additive or synergistic, the combination index (CI) was determined using the Chou-Talalay method (CI=1, additive effect; CI<1, synergism; CI>1, antagonism) [36]. We found that ruxolitinib can synergistically increase the anti-tumor activity of paclitaxel in both OVCAR-8 and MDAH2774 cells (Table 2, Figure 3 and 4). The effect of ruxolitinib on the anti-tumor activity of other chemotherapy agents was dependent on the cell-type. For example, the combination of ruxolitinib and ciaplatin or carboplatin is synergistic in OVCAR-8 cells, but not in MDAH2774. The combination of ruxolitinib and doxorubicin or topotecan is not synergistic in both OVCAR-8 and MDAH 2774 cells. To determine the optimal ruxolitinib:paclitaxel molar ratio, OVCAR-8 cells were incubated with ruxolitinib (fixed at 40 μM) and paclitaxel (20 nM to 160 nM) at various paclitaxel:ruxolitinib molar ratios (1:250, 1:500, 1:1000 and 1:2000) (Figure 5A). The combination treatment produced a synergism at each molar ratio (Table 3); however, the 1:1000 molar ratio produced stronger synergy and a lower IC_50_ for both agents in OVCAR-8 cells. To understand whether anti-tumor effect of paclitaxel can also be enhanced by other JAK/STAT3 inhibitors, AZD1480, another JAK/STAT3 inhibitor, was combined with paclitaxel in MDAH2774 cells. The combined treatment is much more effective than either alone with CI<1 (Figure 5B and 5C). Taken together, our results demonstrated that blocking JAK/STAT3 pathway can synergistically enhance anti-tumor activity of paclitaxel in ovarian cancer cells.

**Table 2:** Interaction between ruxolitinib and chemotherapy agents in human ovarian cancer cells

Cells	Combination index (CI)														
	Paclitaxel			Carboplatin			Cisplatin			Doxorubicin			Topotecan		
	ED50	ED75	ED90	ED50	ED75	ED90	ED50	ED75	ED90	ED50	ED75	ED90	ED50	ED75	ED90
**OVCAR-8**	1.01	0.93	0.87	1.06	0.85	0.69	0.85	0.48	0.33	1.38	1.21	1.13	1.26	1.22	1.19
**MDAH2774**	0.96	0.86	0.83	1.24	1.15	1.08	1.08	1.01	0.99	1.30	1.12	0.99	1.15	1.05	0.96

**Table 3:** Synergistic interaction between ruxolitinib and paclitaxel in variety of molar ratios in OVCAR-8 cells

Paclitaxel: Ruxolitinib	Combination index (CI)			IC50	
	ED50	ED75	ED90	Ruxolitinib (μM)	Paclitaxel (nM)
**1:250**	1.00	0.93	0.86	3.70	14.8
**1:500**	1.01	0.93	0.87	5.66	11.32
**1:1000**	1.06	0.96	0.87	7.93	7.93
**1:2000**	1.12	1.07	1.01	11.02	5.51

### Effects of combination treatment with ruxolitinib and paclitaxel on apoptosis

**Figure 6:**
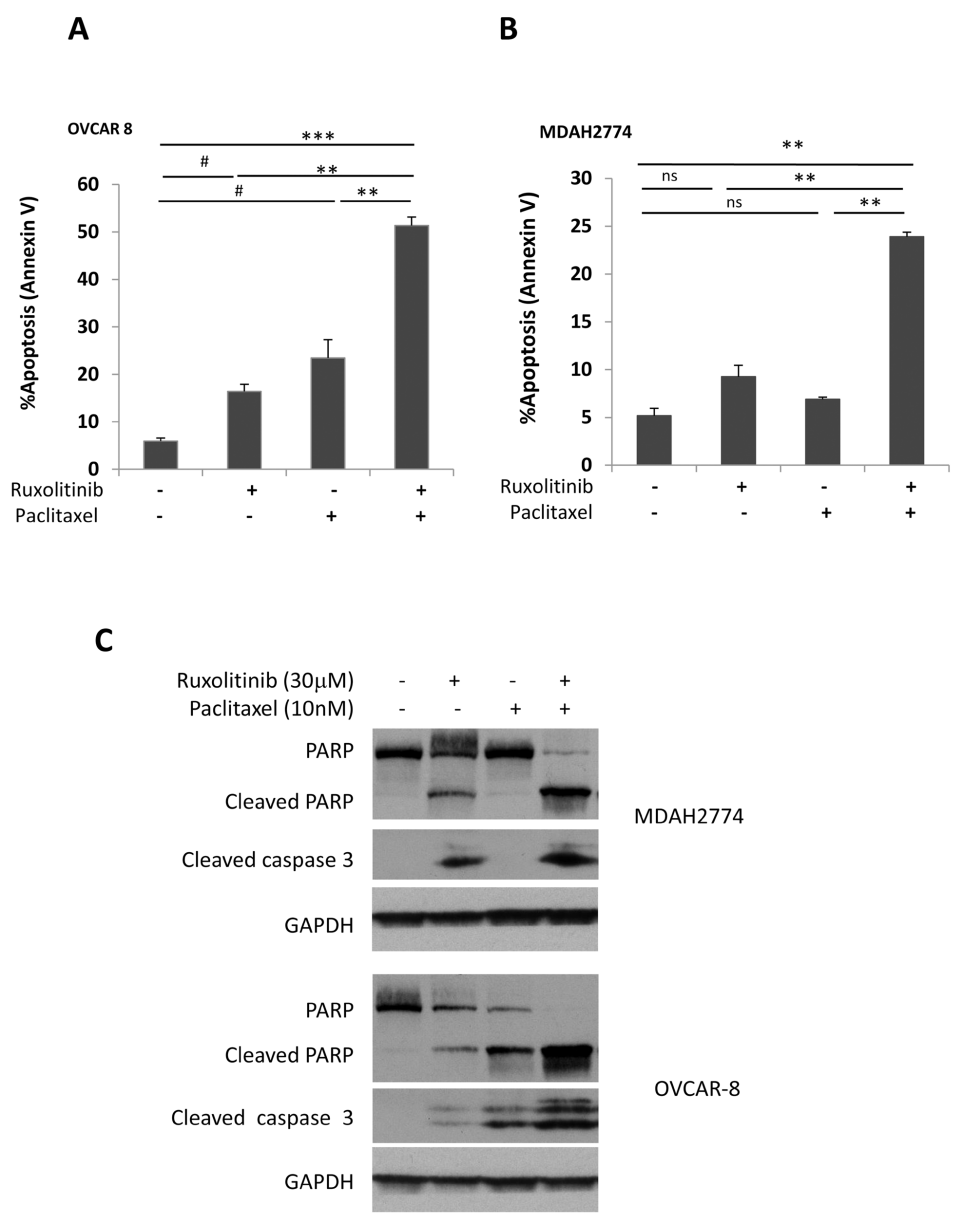
Ruxolitinib enhanced paclitaxel-induced apoptosis in human ovarian cancer cells **(A)** OVCAR-8 and **(B)** MDAH2774 cells were treated with ruxolitinib (30μM), paclitaxel (10nM) either alone or together, for 48 h. Apoptosis was determined by flow cytometry using annexin V and PI staining (A&B) or by cleaved poly-ADP ribose polymerase (PARP) and cleaved caspase-3 by Western blot **(C)**. ns: not significant; ^#^*P<*0.05, ruxolitinib alone or paclitaxel alone vs control; ^**^P<0.005; ^***^P<0.0005, ruxolitinib alone, paclitaxel alone or control *vs* combination.

Next, we determined whether the synergistic effect of ruxolitinib and paclitaxel is due to induction of apoptosis. OVCAR-8 and MDAH2774 cells were treated with ruxolitinib and paclitaxel either alone or in combination for 48 h, and the number of apoptotic cells was determined by annexin V staining. Paclitaxel-induced apoptosis increased from 23.48% to 51.33% in OVCAR-8 cells and from 6.91% to 23.92% in MDAH2774 cells when combined with ruxolitinib (Figure 6A). Consistent with the annexin V staining results, more cleaved caspase 3 and cleaved poly-ADP ribose polymerase (PARP) were generated in cells that were treated with both ruxolitinib and paclitaxel (Figure 6B). These results indicate that inhibition of the JAK/STAT3 pathway could enhance the sensitivity of these human ovarian cancer cells to paclitaxel by promoting apoptosis.

### Effect of combination treatment on ovarian cancer growth in mice

**Figure 7:**
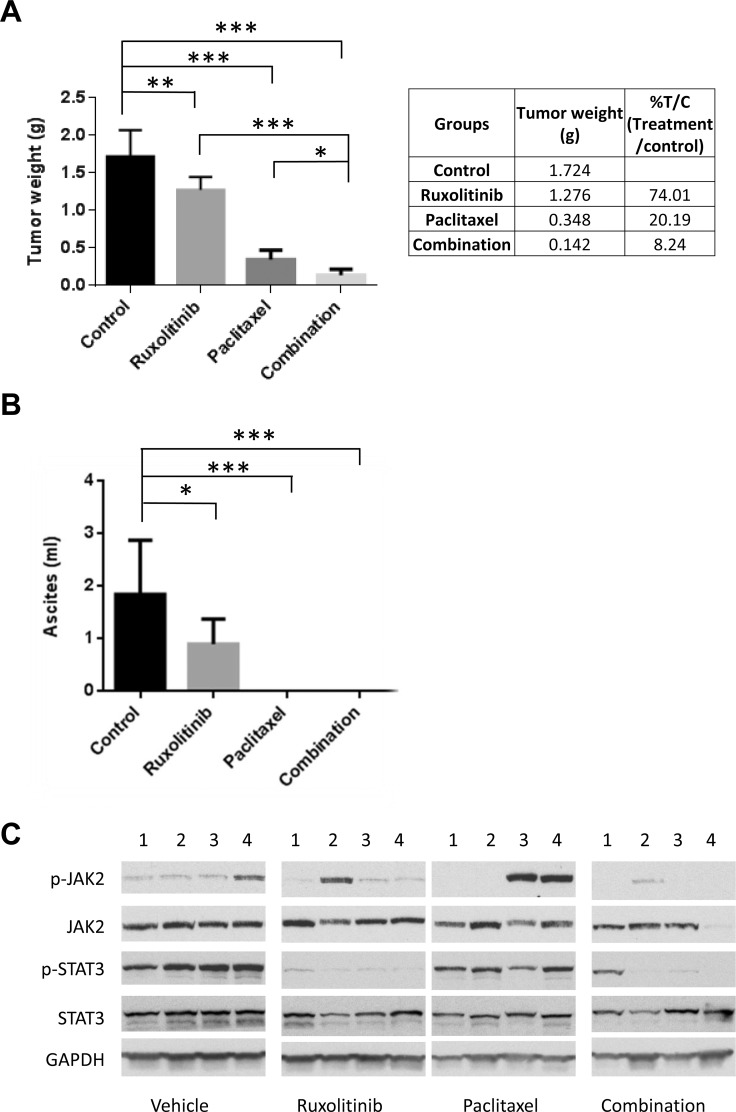
Ruxolitinib enhanced anti-tumor activity of paclitaxel in mice OVCAR-8-ip-luc cells were implanted into peritoneal cavity of NSG mice. Mice were randomized into 4 groups and treated with control chow, chow supplemented with ruxolitinib (2000mg/kg), paclitaxel (10mg/kg; via i.p. injection every 4 days for 3 times), or the combination of both. Mice were euthanized four weeks later. **(A)** Large tumor and small tumor nodules throughout the peritoneal cavity were excised and weighed. **(B)** Ascites were collected, and their volumes were measured. n=4-9, ^*^P<0.05; ^**^P<0.005; ^***^P<0.0005. **(C)** Ruxolitinib inhibited the activation of STAT3 in the tumors. Whole tumor lysates were prepared and analyzed for expression of p-STAT3, p-JAK2, total STAT3, and total JAK2 by Western blot.

Next, we investigated whether the combination treatment could suppress tumor growth more effectively than either treatment alone in a mouse tumor model that represents late stage ovarian cancer with peritoneal metastasis and ascites formation. OVCAR-8-ip-Luc cells were generated and used for this model. OVCAR-8-ip-Luc is a highly metastatic human ovarian cancer cell derived from OVCAR-8 cells by selecting for a peritoneal metastatic phenotype in the mice (Materials and Methods). One week following i.p. injection of OVCAR-8-ip-Luc, mice were randomized into four groups and treated with vehicle control, ruxolitinib, paclitaxel, or paclitaxel plus ruxolitinib. Ruxolitinib was given orally in chow formulation (2g ruxolitinib in 1kg chow), which has been successfully used in a number of studies [37–41]. The serum level of ruxolitinib was shown to fall within the range achieved in humans. Both food consumption and body weight were monitored over the course of treatment and they were comparable between mice with ruxolitinib chow and mice with control chow. Treatment with ruxolitinib chow alone decreased tumor weight from 1.724g to 1.276g, and treatment with paclitaxel alone decreased tumor weight from 1.724g to 0.348g. However, the combination treatment further decreased the tumor weight to 0.142g (Figure 7A), suggesting that the combination treatment was more effective than any single treatment.

To investigate the molecular changes in the tumors upon treatment, tumor tissue lysates were analyzed for the expression of p-STAT3 and p-JAK2 by Western blot. Phosphorylation of STAT3 was blocked in the presence of ruxolitinib, either alone or in combination with paclitaxel (Figure 7C). Overall, these results support a synergistic effect of ruxolitinib and paclitaxel on ovarian cancer cell growth and survival both *in vitro* and *in vivo*.

### Dual treatment of ruxolitinib and paclitaxel led to the reduction of MCL-1 expression in ovarian cancer cells

**Figure 8:**
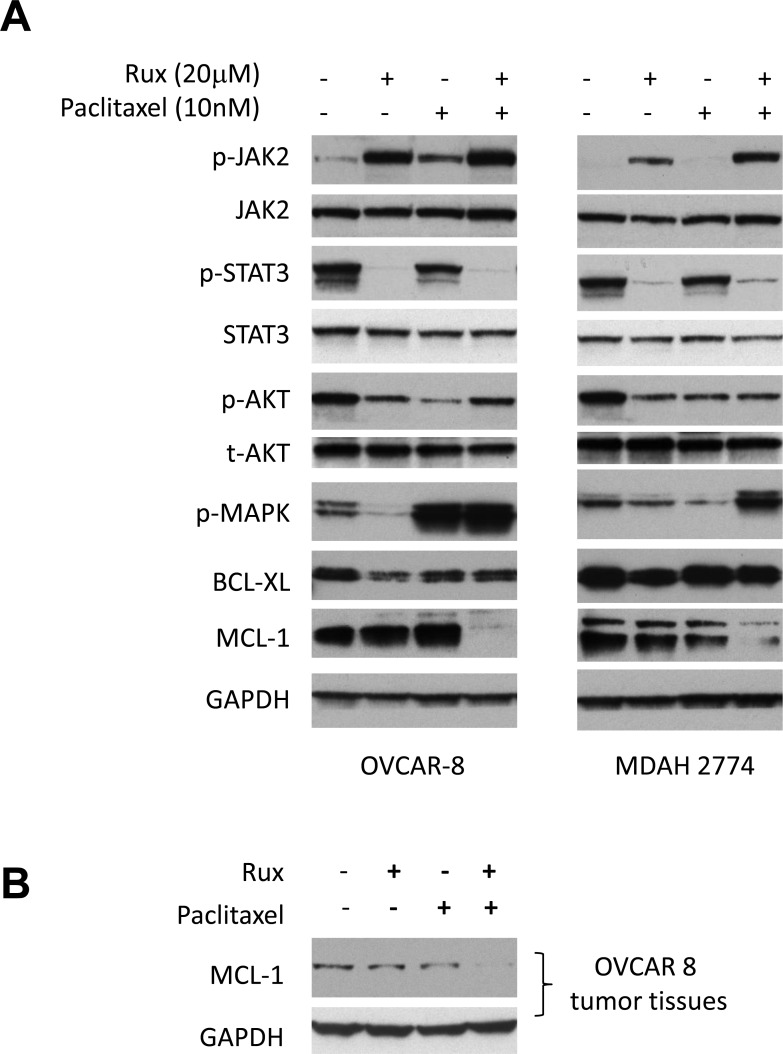
Combined treatment of ruxolitinib and paclitaxel led to the reduction of MCL-1 expression **(A)** OVCAR-8 and MDAH2774 cells were treated with ruxolitinib (20μM), paclitaxel (10nM) or the combination for 24 h. Whole cells were collected and determined for the change of STAT3, AKT and ERK pathways and expression of BCL-XL and MCL-1 by Western blot. **(B)** Whole tumor lysates from the respective groups (4-8) were pooled and analyzed for expression of MCL-1 by Western blot.

To understand the molecular mechanism underlying this synergistic effect, we investigated the effect of combined treatment on the expression of proteins involved in cell signaling and cell survival. A number of signaling pathways, including MAPK/ERK, PI3K/AKT and JAK/STAT3 pathways, are constitutively activated and play important roles in the growth and progression of ovarian cancer. To study the effect of ruxolitinib and paclitaxel on these signaling pathways, OVCAR-8 and MDAH2774 cells were treated with ruxolitinib and paclitaxel either alone or in combination for 24 hours, and tested for the expression of p-STAT3, p-AKT, p-ERK, as well as BCL-XL and MCL-1, two important STAT3 downstream proteins, by Western blot. As shown in Figure 8A, the combination of ruxolitinib and paclitaxel caused a significantly reduction of MCL-1, an important pro-survival protein. Consistent with this result, MCL-1 expression was also reduced in the tumor tissues that were treated with both ruxolitinib and paclitaxel (Figure 8B). Taken together, our results suggested that the synergistic effect by combining ruxolitinib and paclitaxel might be mediated via reducing pro-survival proteins, such as MCL-1 and promoting apoptosis. However, additional study is needed to further understand the mechanism underlying the synergy.

## DISCUSSION

The JAK/STAT3 pathway has emerged as an attractive target for cancer treatment [42, 43]. In this study, we investigated the therapeutic potential of the JAK1/JAK2 inhibitor ruxolitinib in ovarian cancer treatment, either alone or in combination with conventional chemotherapy agents. Ruxolitinib has been previously FDA-approved for the treatment of MF [30]. Our results demonstrate that ruxolitinib, either alone or in combination with paclitaxel, can significantly inhibit STAT3 activation and ovarian tumor growth both in cells and in a mouse model.

Drug resistance remains one of the major challenges in the treatment of ovarian cancer [24, 44, 45]. Most patients eventually develop resistance to treatment despite high initial response rates to first line platinum-taxane treatment. Currently available agents used to treat drug resistant patients include paclitaxel, pegylated liposomal doxorubicin, and topotecan [44]. However, the response rate is in the 10-15% range, and the overall survival rate is only about 12 months. To improve the treatment for drug resistant ovarian cancer, alternative approaches have been developed, including the use of novel cytotoxic reagents and the combination of chemotherapy with targeted agents. Previous studies have shown that constitutive activation of STAT3 confers resistance to platinum and paclitaxel induced apoptosis in ovarian cancer [20, 27]. Our study demonstrates that ruxolitinib, an FDA-approved JAK inhibitor, can potently enhance the anti-tumor activity of paclitaxel in ovarian cancer both *in vitro* and *in vivo*. Although the mechanism of this synergistic interaction is currently unclear, our preliminary results suggest that suppression of STAT3 activation may involve down-regulation of anti-apoptosis proteins, such as MCL-1.

To disrupt STAT3 signaling and activity, various approaches have been explored to identify small molecule inhibitors targeting members of the STAT3 signaling pathway [46]. Although a number of inhibitors have been developed to directly inhibit STAT3 activity in preclinical studies, none of these direct inhibitors are currently in clinical studies for cancer treatment. In contrast, targeting the upstream kinase activity of STAT3 has led to the discovery of several inhibitors in clinical studies, including ruxolitinib (INCB018424), tofacitinib (CP-690550), fedratinin (TG101348), pacritinib (NCT01773187, SB1518), momelotinib (CYT387, NCT00935987), and baricitinib (INCB 028050) [46–53]. However, off-target toxicity is still a concern due to the conserved ATP binding site in the kinase family and the ability of kinase to activate more than one target. Ruxolitinib is the only JAK1/JAK2 inhibitor FDA-approved for the treatment of MF [30]. The therapeutic application of ruxolitinib in solid tumors is currently in clinical study in several solid tumors including ovarian, metastatic breast, and pancreatic cancers [34, 54]. The combined treatment of ruxolitinib with paclitaxel and carboplatin is currently under clinical evaluation in human ovarian cancer. However, little preclinical information is available about the effect of ruxolitinib in ovarian cancer growth. Our results demonstrated for the first time that the combination of ruxolitinib with paclitaxel is more effective against ovarian cancer growth than either agent alone, suggesting that ruxolitinib may be used to improve drug sensitivity and reduce undesirable side effects associated with conventional chemotherapy.

Taken together, our findings provide a valuable preclinical foundation for clinical trials with ruxolitinib either as a single agent or in combination with paclitaxel for the treatment of recurrent and advanced ovarian cancer.

## MATERIALS AND METHODS

### Reagents

Ruxolitinib was kindly provided by Incyte Inc. AZD1480 was kindly provided by AstraZeneca. Antibodies against p-STAT3 (Y705), STAT3, p-JAK2 (Y1007/1008), JAK2, p-AKT, p-ERK, BCL-XL, PARP, Caspase 3, and GAPDH were obtained from Cell Signaling Technology (Danvers, MA). Antibodies against AKT and MCL-1 were from Santa Cruz Biotechnology (Dallas, TX). The antibody against actin was obtained from Sigma (St. Louis, MO).

### Cell culture

SKOV3 and MDAH2774 cells were obtained from ATCC. The OVCAR-8 cells were obtained from the National Cancer Institute. SKOV3 and MDAH2774 cells were cultured in DMEM medium. OVCAR-8 cells were cultured in RPMI1640 medium. Culture media were supplemented with 10% FBS and 1% penicillin/streptomycin (P/S). All cells were grown in 5% (v/v) CO_2_ at 37°C.

To minimize mouse-to-mouse variation in tumor formation of OVCAR-8 cells, a subline of OVCAR-8, OVCAR-8-ip, was generated. To do this, parental OVCAR-8 cells were inoculated into the peritoneal cavity of NOD/SCID/IL2R gamma null (NSG) mice and allowed to form tumor. The mouse that had the most ascites and peritoneal metastasis at earliest time point was used to isolate tumor cells from the ascites. The isolated tumor cells, called OVCAR-8-ip, produced ascites and formed peritoneal tumors with much less variation between mice when these cells were implanted into mice. To monitor peritoneal tumor growth using imaging, OVCAR-8-ip cells were stably transfected with CMV-p:EGFP-ffluc pHIV7 (a gift from Christine Brown at City of Hope) as previously described to create OVCAR-8-ip-Luc cells [55].

### Cell viability assays

Cells were plated in 96-well plate format in 100 μl growth medium. To ensure that all cells had similar cell confluence when placed, we placed MDAH2774 at 7000 cells per well and other cells at 4000 per well. Cells were treated with DMSO or drugs the next day at the indicated concentrations and incubated for an additional 1 to 3 days. Viable cells were determined either by MTS assay (Promega, Madison, WI, USA) or acid phosphatase assay as described previously [56]. The IC_50_ was determined using Calcusyn (Biosoft, Ferguson, MO).

### Determination of combination index (CI)

The combination index (CI) was determined using the Chou-Talalay method [36] using Calcusyn (Biosoft, MO).

### Annexin V staining

Apoptosis was measured using an Annexin V Apoptosis Detection Kit (BD bioscience). Briefly, ovarian cancer cells were treated with ruxolitinib, paclitaxel, or both. After 48 h, floating and attached cells were collected and stained with FITC-Annexin V and PI (propidium iodide). The staining intensity was then quantified using fluorescence-activated cell sorting (FACS).

### Western blot analysis

Western blots were performed as described previously [57]. Cells were grown in complete medium overnight and treated with DMSO or drugs at various concentrations for 24 h. Cells were washed in cold PBS and lysed in RIPA lysis buffer (Thermo Scientific) containing Halt Protease and Phosphatase Inhibitor Cocktail (Thermo Scientific). Proteins were quantified using BCA protein assay reagent (Thermo Scientific). Equal amounts of protein were separated by SDS-polyacrylamide gel electrophoresis, transferred to polyvinylidene fluoride membranes, and incubated with total and phosphorylated protein-specific antibodies. Binding of the primary antibody was detected using a horseradish peroxidase (HRP)-conjugated secondary antibody and chemiluminescent substrates (Thermo Scientific).

### Animal models

All animal studies were carried out under protocols approved by the City of Hope Institutional Animal Care and Use Committee (IACUC) in accordance with guidelines of the association for Assessment and Accreditation of Laboratory Animal Care.

OVCAR-8-ip-Luc cells (5×10^6^ in 100 μl) were inoculated into the peritoneal cavity of 6- to 8- week-old female NSG mice. Starting one week after inoculation, mice were treated with control, ruxolitinib, paclitaxel (10mg/kg via i.p. injection, every 4 days for total 3 times), or combination of both. Ruxolitinib was given orally in chow formulation (2g ruxolitinib in 1kg chow) as described previously (kindly provided by Incyte) [37–41]. We monitor food consumption during period of treatment to ensure comparable amount of food was taken between mice with ruxolitinib chow and mice with control chow. Body weight was monitored weekly as an indicator of drug-induced toxicity and overall health of the mice. The mice were monitored for ascites production and any adverse effects. Mice were euthanized 25 days after cell inoculation. Visible tumor nodules were excised and weighed, and the ascites fluid was collected and measured for the volume.

### Statistical analysis

Data are presented as mean ± S.D. Student's *t*-test was used to compare the means of two groups. Experiments were carried out in triplicate or more. P < 0.05 was considered statistically significant.
